# Serum uromodulin is associated with the severity of clinicopathological findings in ANCA-associated glomerulonephritis

**DOI:** 10.1371/journal.pone.0224690

**Published:** 2019-11-14

**Authors:** Shohei Tachibana, Masayuki Iyoda, Taihei Suzuki, Nobuhiro Kanazawa, Ken Iseri, Yukihiro Wada, Kei Matsumoto, Takanori Shibata

**Affiliations:** Division of Nephrology, Department of Medicine, Showa University School of Medicine, Tokyo, Japan; University of Missippi Medical Center, UNITED STATES

## Abstract

**Background:**

Uromodulin (UMOD), also known as Tamm-Horsfall protein, is a kidney-specific protein expressed by epithelial cells lining the thick ascending limb of the loop of Henle. In the current study, we aimed to clarify the clinical significance of UMOD in ANCA-associated glomerulonephritis (AAG).

**Materials and methods:**

Sixty-one biopsy-proven AAG patients were included in this study. UMOD was measured using ELISA. The relationships between serum UMOD (sUMOD) levels and various clinicopathological findings were evaluated.

**Results:**

AAG was classified into four categories (focal, crescentic, mixed, and sclerotic). In addition, tubulointerstitial lesions were classified as mild, moderate, and severe. The levels of sUMOD and urinary UMOD (uUMOD) were correlated with each other. A negative correlation between sUMOD levels and serum Cr levels, and positive correlation between sUMOD levels and eGFR were found. Patients in the high sUMOD group were associated with low serum Cr levels, focal classification, and mild tubulointerstitial injury compared to the low sUMOD group. Comparing the characteristics among histopathological classes, patients in the focal class had the best renal function and the highest levels of uUMOD/Cr and sUMOD. The focal class had significantly better renal survival compared with the severe histopathological classes (crescentic, mixed, and sclerotic). In univariate logistic regression analyses, prognostic factors for severe histopathological classes were low uUMOD/Cr, high serum Cr, and low sUMOD. Multivariate analyses revealed that low sUMOD predicted severe histopathological classes independent of serum Cr. The mean levels of sUMOD were significantly different between the focal class and severe histopathological classes, with a sensitivity of 70.6% and specificity of 90.0% (cut-off 143 ng/ml, AUC 0.80) by ROC curves.

**Conclusion:**

Low sUMOD levels were associated with severe clinicopathological findings and might be considered as a risk factor for end stage renal disease in AAG.

## Introduction

Tamm-Horsfall protein (THP), which was identified by Tamm and Horsfall in 1950, is the most abundant protein excreted in the urine by epithelial cells lining the thick ascending limb (TAL) of the loop of Henle; the protein has been reported to interact with and inhibit viral hemagglutination [1, 2]. In 1985, Muchmore and Decker reported that uromodulin (UMOD), a 85 kDa glycoprotein isolated from the urine of pregnant women, had the ability to inhibit antigen-induced T-cell proliferation and monocyte cytotoxicity *in vitro* [3]. Later, THP and UMOD were identified as the same protein based on sequence analysis [4]. UMOD has been reported to have a variety of physiologic functions, such as inhibiting urinary tract infections [5], promoting urinary cast formation [6, 7], and regulating the activity of the renal outer medullary potassium channel (ROMK) and of the sodium-potassium-chloride transporter (NKCC2) [8, 9]. In addition, UMOD gene mutations cause UMOD-associated kidney disease (UAKD), and polymorphisms in the UMOD gene are strongly linked to chronic kidney disease (CKD) [10].

In patients with CKD of various etiologies, the urinary excretion levels of UMOD are usually decreased and closely correlated with changes in the estimated glomerular filtration rate (eGFR) [11]. Lower urinary UMOD (uUMOD) levels are associated with higher odds for AKI after cardiac surgery [12]. In patients with IgA nephropathy, low levels of uUMOD are correlated with eGFR decline and are associated with the severity of tubulointerstitial injury [13]. On the other hand, UMOD is also present in serum by way of leaking from the basolateral side of epithelial cells of TAL. Several studies have shown that serum UMOD (sUMOD) levels are also correlated with renal function and decline along with the progression of CKD [14–16] and AKI [17].

In the current study, we investigated sUMOD levels in anti-neutrophil cytoplasmic antibody (ANCA)-associated glomerulonephritis (AAG) patients. AAG frequently occurs as rapidly progressive glomerulonephritis (RPGN) over its clinical course and causes renal death. In 2010, a renal histopathological classification of AAG in European patients was proposed, and the four general categories (focal, crescentic, mixed, and sclerotic) significantly correspond to renal survival [18]. This histological classification has been validated for various ethnic groups and countries, and its usefulness has been established [19–21]. Concerning renal prognosis, the focal class has good prognosis, while the sclerotic class has poor prognosis, findings that have been repeatedly reproduced [19–21]. Although renal biopsies have an essential role in the diagnosis and treatment of various kidney diseases, this invasive procedure is sometimes associated with bleeding, which can be lethal, especially among elderly patients. Thus, biomarkers that allow clinicians to predict renal survival and histological features without requiring renal biopsies are necessary in AAG, as it is by far the most common cause of RPGN in the elderly population. In this study, we evaluated whether sUMOD can predict renal survival and is correlated with clinicopathological findings in patients with AAG.

## Materials and methods

### Patients and samples

This study was approved by the Ethics Committee of Showa University Hospital in Tokyo, Japan (No. 2252), and patient data were anonymously used under consideration of the latest version of the Helsinki Declaration of human research ethics. Sixty-one patients with AAG in accordance with the Chapel Hill consensus criteria (CHCC) [22], treated at Showa University Hospital between April 2003 and April 2017, were selected. Thirty-five of the renal biopsy examinations were performed before the initiation of immunosuppressive therapy. Renal survival data were available for 54 of 61 patients. Renal survival was analyzed at onset, 1 year, and 12 years after renal biopsy in available patients (54 at onset, 46 at 1 year, and 6 at 12 years).

Blood samples at the time of renal biopsy were collected from all patients (n = 61). Urine samples at the time of renal biopsy were collected after 2008 (n = 40). Blood samples and urine samples were stored at -80°C until use.

### UMOD, α1MG, β2MG, and NAG measurements

sUMOD and uUMOD concentrations were measured with a commercially available enzyme-linked immunosorbent assay (ELISA) kit (BioVendor, Candler, NC). The urinary levels of α1-microglobulin (MG), β2-MG, and N-acetyl-β-D-glucosaminidase (NAG) were measured in the clinical laboratory at Showa University Hospital (1993–2015) or BML, Inc. (from 2016). All urinary measurements were normalized individually to urinary creatinine (Cr) levels.

### Renal biopsy

Renal biopsy specimens were divided and processed for light, immunofluorescence (IF), and electron microscopy (EM) analysis. Sections were made from formalin-fixed, paraffin-embedded tissue and stained by hematoxylin and eosin (H-E), periodic-acid Schiff (PAS), periodic acid methenamine silver (PAM), and Masson trichrome stains for light microscopy. For IF, renal tissues were snap frozen in liquid nitrogen, and the glomerular deposits of IgG, IgA, IgM, C3, C4, C1q, fibrinogen, κ-light chain, and λ-light chain in the biopsy specimens were examined. EM was performed at the Department of Pathology, Showa University School of Medicine (1993–2015) or Pathology & Cytology Laboratories (PCL) Japan, Inc. (from 2016).

### Pathological classification of AAG

**Fig 1:**
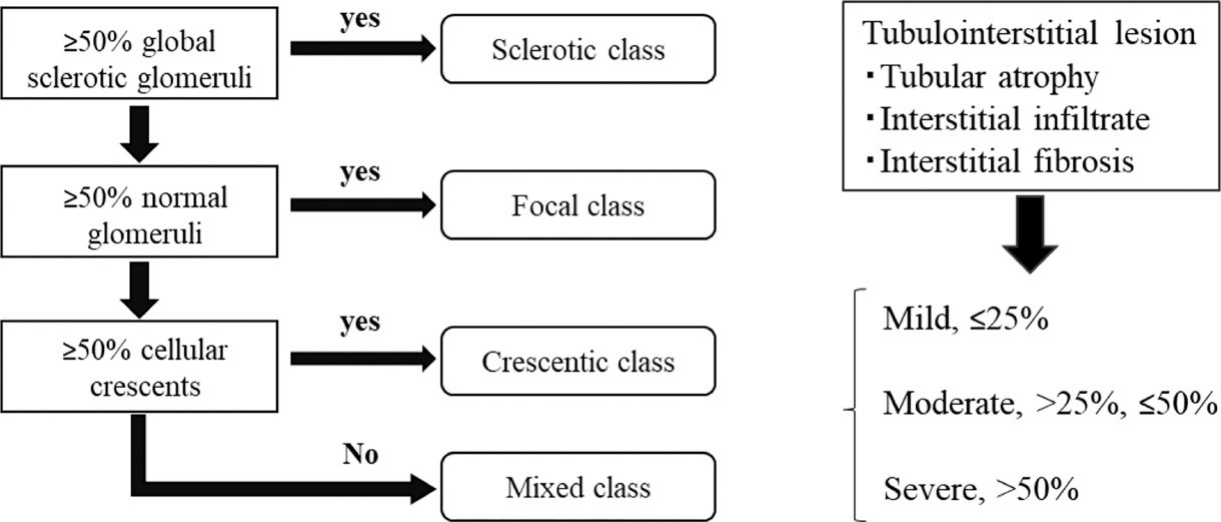
Schematic representation of pathological categorizations based on glomerular lesions and semiquantitative analysis of tubulointerstitial injury in ANCA-associated glomerulonephritis.

The pathological categorization of AAG based on glomerular lesions was performed for specimens that included at least 10 glomeruli as previously described by Berden’s group [18]: focal ≥50% normal glomeruli; crescent ≥50% of glomeruli with cellular crescents; sclerotic ≥50% of glomeruli with global sclerosis; and mixed <50% normal, <50% crescentic, <50% globally sclerotic glomeruli (Fig 1).

### Semiquantitative analysis for tubulointerstitial injury

The extent of tubulointerstitial injury was evaluated by counting the percentage of area with interstitial inflammatory cell infiltration, interstitial fibrosis, and tubular atrophy per field of cortex. The extent of tubulointerstitial injury was classified as mild (<25% of area injured), moderate (25 to 50% of area injured), or severe (>50% of area injured) (Fig 1).

### Definition of end stage renal disease and calculation of estimated glomerular filtration rate

End stage renal disease (ESRD) was defined as the need for dialysis (hemodialysis or peritoneal dialysis). The estimated glomerular filtration rate (eGFR) was calculated using the Cockcroft-Gault formula.

### Statistical analysis

Data were expressed as means ± standard deviation (SD) or medians (range from the 25th to the 75th percentile) for continuous variables and as numbers (percentage) for nominal variables. Distributions were tested for normality with Shapiro-Wilk’s test. Comparisons between two groups for normally distributed continuous variables were performed using Student’s *t* test, and the Wilcoxon rank sum test was used for non-normally distributed continuous variables. Comparisons between four groups for normally distributed continuous variables were performed using one-way analysis of variance (ANOVA), and the Kruskal-Wallis test was used for non-normally distributed continuous variables. Fisher’s exact test and the chi-squared test were used to compare nominal variables between groups. The correlation between several parameters was analyzed by Spearman’s rank coefficient of correlation. Renal survival was analyzed using the Kaplan-Meier method and Log-rank test. Univariate and multivariate Cox regression analyses were performed to identify predictors of renal survival. Results are expressed as hazard ratios (HR) with 95% confidence intervals (95% CI). To evaluate the association of severe histopathological classes (crescentic, mixed, and sclerotic) with clinical parameters, univariate and multivariate logistic regression analyses were performed. Results are expressed as odds ratios (OR) with 95% CI. Receiver operating characteristics curves (ROC) were used to present diagnostic utility; area under curve (AUC) values with sensitivity and specificity were determined. All statistical analyses were performed by JMP Pro ver. 11.0 for Windows. For all tests, the level of significance was set at p<0.05.

## Results

### Baseline clinical characteristics

The clinical characteristics of the patients are shown in Table 1. Among the 61 patients with AAG, 52% were male, and the median age at the time of diagnosis was 70 years. The median urinary protein level and the mean prevalence of hematuria were 0.7 g/day and 77%, respectively. The median serum Cr and eGFR were 1.3 mg/dl and 34.1 ml/min/1.73 m^2^, respectively. According to the CHCC classification, 55 had microscopic polyangiitis (MPA), 5 had granulomatosis with polyangiitis (GPA), 1 had eosinophilic granulomatosis with polyangiitis (EGPA), and all were positive for myeloperoxidase-ANCA (MPO-ANCA). Twenty-five (41%) patients had a history of hypertension, and 6 (10%) had diabetes mellitus.

**Table 1:** Baseline clinical characteristics.

Characteristic	Total (n = 61)	Serum uromodulin ≦156 (ng/ml)(n = 31)	Serum uromodulin >156 (ng/ml)(n = 30)	P Value
Age (years)	70 (58, 74)	68 (58, 73)	71 (57, 75)	0.65
Men, n (%)	32 (52)	17 (55)	12 (40)	0.31
BMI (Kg/m^2^)	21.7 (19.5, 24.0)	21.6 (18.8, 23.9)	21.8 (19.6, 24.0)	0.56
SBP (mmHg)^a^	128.5 ± 20.4	127.7 ± 21.9	129.3 ± 19.0	0.77
DBP (mmHg)^a^	72.4 ± 12.8	71.7 ± 13.5	73.2 ± 12.3	0.67
Proteinuria (g/g)	0.7 (0.3, 1.8)	1.1 (0.4, 1.9)	0.5 (0.2, 1.5)	0.13
Hematuria, n (%)	47 (77)	24 (77)	23 (77)	1.00
NAG index (U/g)	20.5 (13.0, 42.2)	20.5 (12.9, 42.4)	20.6 (13.8, 39.0)	0.91
Urinary β2-MG/Cr (mg/g)	5.4 (0.7, 22.9)	9.1 (0.6, 32.7)	4.9 (0.7, 20.9)	0.65
Urinary Uromodulin/Cr (mg/g)^b^	135.8 (47.7, 264.0)	52.9 (35.1, 168.3)	165.3 (113.5, 323.3)	0.021
Hb (g/dl)	9.5 (8.8, 11.3)	9.5 (8.7, 10.2)	9.9 (8.9, 11.8)	0.17
TP (g/dl)^a^	6.7 ± 0.84	6.8 ± 0.7	6.6 ± 1.0	0.28
Alb (g/dl)	3.0 (2.3, 3.5)	3.2 (2.3, 3.6)	2.9 (2.4, 3.5)	0.67
BUN (mg/dl)	25.4 (19.1, 39.1)	31.7 (22.3, 41.9)	21.5 (17.2, 31.4)	0.018
Cr (mg/dl)	1.3 (0.9, 2.4)	1.8 (1.2, 3.4)	1.2 (0.9, 1.4)	0.0073
eGFR (ml/min/1.73m^2^)	34.1 (19.6, 48.3)	25.3 (14.3, 38.6)	39.8 (30.3, 49.5)	0.0081
Serum uromodulin (ng/ml)	155.1 (116.8, 219.8)	117.8 (70.7, 134.9)	219.8 (193.9, 249.8)	< .0001
CRP (mg/dl)	1.2 (0.2, 5.2)	1.0 (0.2, 9.2)	1.3 (0.2, 4.8)	0.75
ANCA type				
MPO-ANCA, n (%)	61 (100)	31 (100)	30 (100)	1.00
MPO-ANCA titer	115.4 (55.1, 462)	98 (49, 395)	115.4 (74.1, 534)	0.44
PR-3-ANCA, n (%)	0 (100)	0 (0)	0 (0)	1.00
Clinical diagnosis, n (%)				
MPA	55 (90)	28 (90)	27 (90)	1.00
GPA	5 (8)	3 (10)	2 (7)	1.00
EGPA	1 (2)	0 (0)	1 (3)	0.49
Hypertension, n (%)	25 (41)	12 (39)	13 (43)	0.79
Diabetes mellitus, n (%)	6 (10)	3 (10)	3 (10)	1.00
Treatment				
Prednisolone	62 (100)	31 (100)	30 (100)	1.00
Methyl predonisolone pulse	41 (66)	16 (52)	24 (77)	0.11
Cyclophosphamide	33 (54)	15 (48)	18 (60)	0.44
Rituximab	5 (8)	4 (13)	1 (3)	0.35
Azathioprine	16 (26)	8 (26)	8 (27)	1.00
Plasmapheresis	5 (8)	4 (13)	1 (3)	0.35

Abbreviations: BMI, body mass index; SBP, systolic blood pressure; DBP, diastolic blood pressure; NAG, N-acetyl-β-D-glucosaminidase; β2-MG, β2-microgloburin; Cr, creatinine; Hb, hemoglobin; TP, total protein; Alb, albumin; BUN, blood urea nitrogen; eGFR, estimated glomerular filtration rate; CRP, C-reactive protein; MPO, myeloperoxidase; ANCA, anti-neutrophil cytoplasmic antibody, MPA, microscopic polyangitis; GPA, granulomatosis with polyangitis; EGPA, eosinophilic granulomotosis with polyangitis.

All values are expressed as n (%) or median (25th, 75th percentailes).

^a^Mean ± SD.

^b^n = 40

Patients were divided into two groups according to their sUMOD levels, a group with sUMOD ≤156 ng/ml (low sUMOD group, n = 31) and a group with sUMOD >156 ng/ml (high sUMOD group, n = 30), in order to investigate whether sUMOD was correlated with clinical parameters and pathological findings (Table 1). BUN and serum Cr levels were significantly higher, and uUMOD/Cr levels and eGFR were significantly lower in the low sUMOD group compared to the high sUMOD group. No other clinical findings were significantly different between the groups.

**Fig 2:**
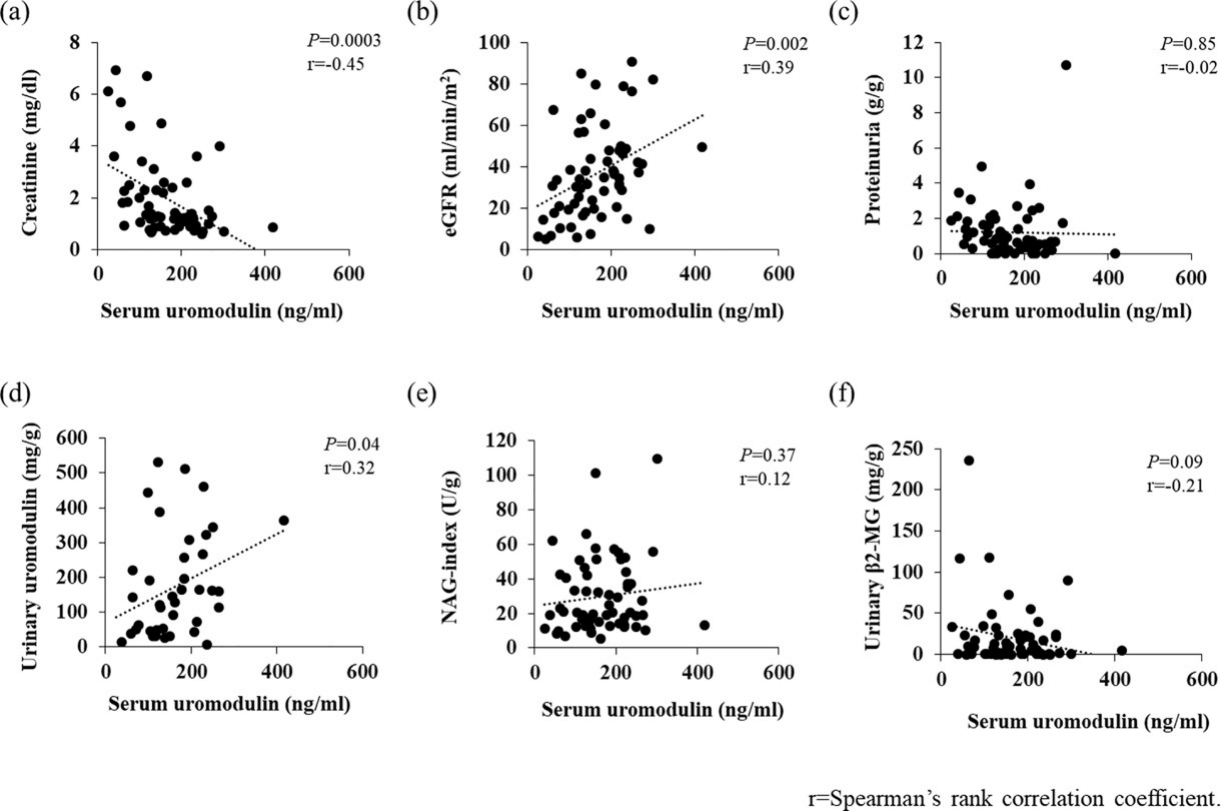
a-f. Correlations between serum uromodulin and creatinine (a), eGFR (b), proteinuria (c), urinary uromodulin /Cr (d), N-acetyl-β-D-glucosaminidase (e), and β2-microgloblin (f).

Analyzing bivariate correlations using Spearman’s correlation coefficient, a negative correlation between sUMOD and serum Cr (r = -0.45) and a positive correlation between sUMOD and eGFR (r = 0.39) were found. sUMOD was also closely correlated with uUMOD/Cr (r = 0.32). However, proteinuria, NAG index, and urinary β2MG were not correlated with sUMOD (Fig 2A–2F).

### Histological differences according to sUMOD levels in patients with AAG

All patients had undergone renal biopsy. Based on the pathological categorization of AAG previously described by Berden’s group [18], we identified 27 (44%) patients in the focal class, 19 (31%) in the crescentic class, 10 (16%) in the mixed class, and 5 (8%) in the sclerotic class (Table 2). In addition, we analyzed tubulointerstitial lesions and identified 21 (34%) patients who were mild, 21 (34%) patients who were moderate, and 19 (31%) patients who were severe (Table 2). There was a significant relationship between sUMOD levels and the percentage of focal class (p<0.001), crescentic class (p<0.05), and mild tubulointerstitial lesions (p<0.05) (Table 2).

**Table 2:** Pathological findings of patients with ANCA-associated glomerulonephritis in each group.

Characteristic	Total (n = 61)	Serum uromodulin ≦156 (ng/ml)(n = 31)	Serum uromodulin >156 (ng/ml)(n = 30)	P Value
Pathological classfication, n (%)				
Focal	27 (44)	6 (19)	21 (70)	< .0001
Crescentic	19 (31)	14 (45)	5 (17)	0.026
Mixed	10 (16)	6 (19)	4 (13)	0.73
Sclerotic	5 (8)	5 (16)	0 (0)	0.052
Tubulointerstitial lesion, n (%)				
mild	21 (34)	6 (19)	15 (50)	0.016
moderate	21 (34)	12 (39)	9 (30)	0.59
severe	19 (31)	13 (42)	6 (20)	0.097

Abbreviations: ANCA, anti-neutrophil cytoplasmic antibody.

All values are expressed as n (%).

### Comparison of clinical findings and renal survival according to renal histopathological categories

Comparing the characteristics among histopathological classes, patients in the focal class had the best renal function including BUN and Cr, the lowest proteinuria, and they had the highest levels of uUMOD/Cr and sUMOD compared with other classes (Table 3). In contrast, patients in the sclerotic class had the worst renal function including BUN and Cr, and they had the lowest sUMOD levels compared with the other classes (Table 3).

**Table 3:** Clinical findings of patients with ANCA-associated glomerulonephritis in each pathological class.

Characteristic	Focal (n = 27)	Crescentic (n = 19)	Mixed (n = 10)	Sclerotic (n = 5)	P Value
Age (years)	72 (61, 75)	64 (55, 72)	70 (61, 78)	67 (52, 74)	0.21
Men, n (%)	12 (44)	8 (42)	6 (60)	3 (60)	0.74
Proteinuria (g/g)	0.4 (0, 0.8)	1.7 (0.7, 2.1)	0.9 (0.3, 1.7)	1.2 (0.6, 3.4)	0.004
Hematuria, n (%)	18 (67)	17 (89)	8 (80)	4 (80)	0.31
Urinary uromodulin/Cr (mg/g)	164.9 (118.1, 339.8)	47.8 (37.6, 167.4)	63.0 (41.9, 310.1)	63.6 (27.3, 443.5)	0.041
Hb (g/dl)	9.3 (8.9, 11.3)	9.4 (8.5, 10.2)	10.5 (9.3, 11.7)	10.2 (7.9, 12.3)	0.43
TP (g/dl)^a^	6.6 ± 0.8	6.6 ± 0.7	7.1 ± 1.1	6.8 ± 0.4	0.34
Alb (g/dl)	2.7 (2.2, 3.3)	2.9 (2.3, 3.4)	3.5 (2.8, 3.9)	3.6 (3.0, 4.0)	0.043
BUN (mg/dl)	21.5 (14.5, 31.2)	34.2 (23.6, 39.5)	24.8 (20.3, 44.3)	40.6 (25.4, 55.5)	0.04
Cr (mg/dl)	1.1 (0.8, 1.3)	1.8 (1.2, 3.6)	1.3 (0.9, 3.2)	2.5 (1.6, 5.4)	0.0007
eGFR (ml/min/1.73m^2^)	43.8 (31.7, 63.1)	25.3 (14.3, 34.1)	36.5 (17.4, 51.6)	19.2 (8.3, 29.5)	0.0005
Serum uromodulin (ng/ml)	204.3 (157.9, 234.7)	121.4 (70.7, 183.3)	147.0 (110.4, 220.5)	77.9 (50.4, 118.0)	0.0002
CRP (mg/dl)	2.1 (0.3, 6.9)	2.3 (0.2, 9.2)	0.5 (0.2, 2.0)	0.2 (0.1, 2.7)	0.11
MPO-ANCA titer (IU/ml)	105 (62, 517)	149 (69, 461)	172 (27, 621)	45 (29, 122)	0.38

Abbreviations: ANCA, anti-neutrophil cytoplasmic antibody; Cr, creatinine; Hb, hemoglobin; TP, total protein; Alb, albumin; BUN, blood urea nitrogen

eGFR, estimated glomerular filtration rate; CRP, C-reactive protein; MPO, myeloperoxidase.

All values are expressed as n (%) or median (25th, 75th percentailes).

^a^Mean ± SD.

**Fig 3:**
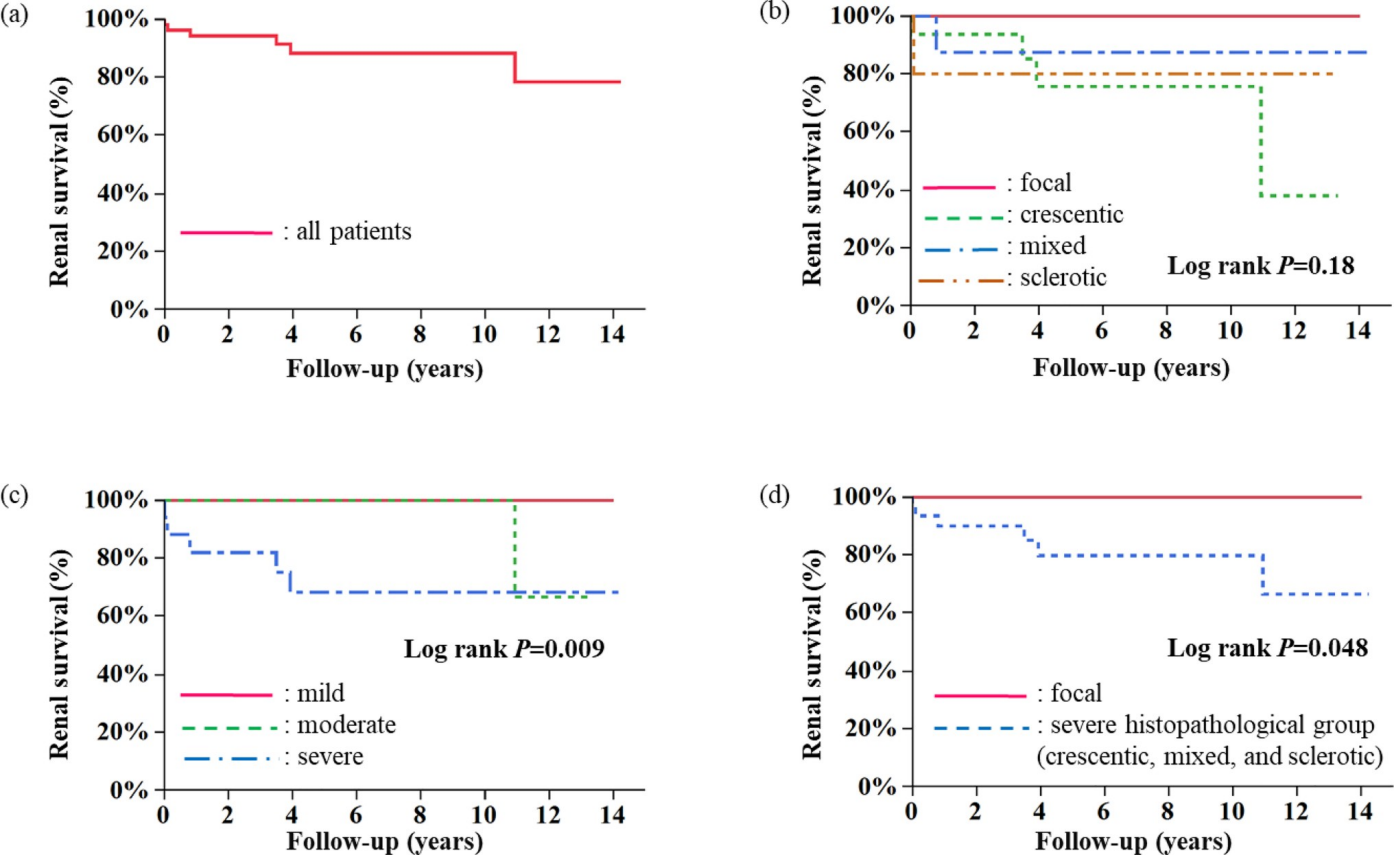
a-d. Renal survival of all patients (a), each histopathological class (focal, crescentic, mixed, and sclerotic) (b), each tubulointerstitial lesion group (mild, moderate, and severe) (c), and focal class and severe histopathological group (crescentic, mixed, and sclerotic) (d).

In Kaplan-Meier analysis, renal survival of all patients and of patients in each group as classified into the four histopathological classes and by severity of tubulointerstitial lesions are shown in Fig 3. Renal survival data were available for 54 of 61 patients. During a follow-up of 5.4 ± 4.3 years, 6 patients (4 patients in crescentic, 1 patient in mixed, and 1 patient in sclerotic class) progressed to ESRD. The cumulative proportions of renal survival at 1 year were 94% for all patients; 100%, 94%, 88%, and 80% for the focal, crescentic, mixed, and sclerotic classes; and 100%, 100%, and 75% for the mild, moderate, and severe tubulointerstitial lesion groups, respectively. The cumulative proportions of renal survival at 12 years were 78% for all patients; 100%, 38%, 88%, and 80% for the focal, crescentic, mixed, and sclerotic classes; and 100%, 67%, and 68% for the mild, moderate, and severe tubulointerstitial lesion groups, respectively. No patients in the focal class or mild tubulointerstitial lesion group developed ESRD (Fig 3B and 3C). Next, we compared renal survival of the focal class with the severe histopathological classes (crescentic, mixed, and sclerotic class). The focal class had significantly better outcomes compared with the severe histopathological classes (P = 0.025) (Fig 3D).

### Prognostic significance of clinical parameters on end-stage renal disease

sUMOD, uUMOD/Cr, and serum Cr were associated with ESRD by univariate Cox regression analysis. However, no significant predictor remained after multivariate Cox regression analysis (Table 4).

**Table 4:** Prognostic significance of clinical parameters on ESRD.

Variable	Univariate Analysis		Multivariate Analysis	
	HR (95% CI)	P Value	HR (95% CI)	P Value
Age (years)	0.97 (0.93 to 1.03)	0.3		
Men	1.08 (0.2 to 6.0)	0.92		
Hypertension	2.84 (0.55 to 20.5)	0.21		
Diabetes mellitus	2.73 (0.14 to 18.7)	0.42		
Proteinuria (g/g)	1.18 (0.87 to 1.45)	0.23		
Urinary uromodulin/Cr/10 (mg/g)	0.83 (0.56 to 0.99)	0.029	0.86 (0.53 to 1.06)	0.15
Hb (g/dl)	0.79 (0.47 to 1.23)	0.31		
Alb (g/dl)	1.07 (0.33 to 3.84)	0.93		
Cr (mg/dl)	1.98 (1.38 to 3.01)	0.004	1.51 (0.85 to 2.76)	0.15
Serum uromodulin/10 (ng/ml)	0.86 (0.73 to 0.98)	0.019	0.95 (0.73 to 1.17)	0.64
CRP (mg/dl)	0.94 (0.71 to 1.10)	0.50		
MPO-ANCA titer (IU/ml)	0.99 (0.99 to 1.00)	0.91		

Abbreviations: ESRD, end-stage renal disease; HR, hazard ratio; CI, confidence interval; Cr, creatinine; Hb, hemoglobin; Alb, albumin; CRP, C-reactive protein; MPO, myeloperoxidase; ANCA, anti-neutrophil cytoplasmic antibody.

Each parameter that was significant in univariable analysis, was analyzed in a multivariable analysis.

### Predictors of severe histopathological class

**Fig 4:**
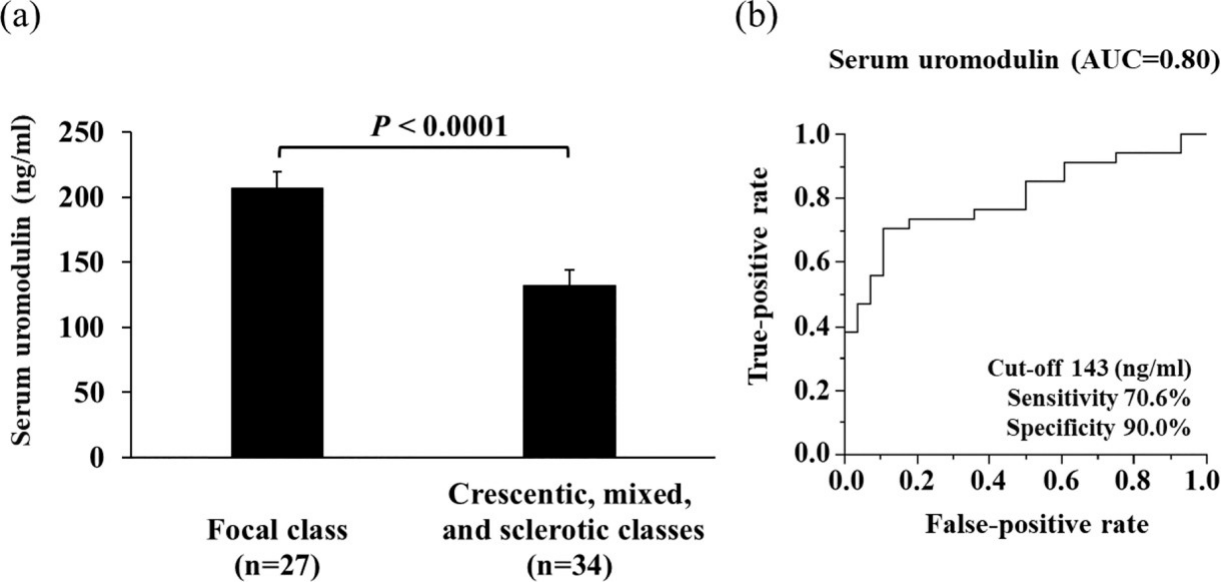
a and b. (a) Serum uromodulin levels in the focal class and the severe histopathological groups (crescentic, mixed, and sclerotic). (b) ROC curve showing the diagnostic utility of serum uromodulin for distinguishing focal class from severe histopathological groups with a sensitivity of 71%, a specificity of 89%, and AUC of 0.80 (cut-off 143 ng/ml).

In univariate logistic regression analyses, clinical parameter prognostic factors for severe histopathological classes were low uUMOD/Cr, high serum Cr, and low sUMOD (Table 5). Multivariate analyses revealed that low sUMOD predicted severe histopathological classes independent of serum Cr. The mean levels of sUMOD were significantly different between the focal class and severe histopathological classes with a sensitivity of 70.6% and specificity of 90.0% (cut-off 143 ng/ml, AUC 0.80) by ROC curves (Fig 4A and 4B).

**Table 5:** Prognostic significance of clinical parameters on sever histopathological classes (crescentic, mixed, and sclerotic).

Variable	Univariate Analysis		Multivariate Analysis	
	HR (95% CI)	P Value	HR (95% CI)	P Value
Age (years)	0.98 (0.94 to 1.03)	0.38		
Men	1.25 (0.45 to 3.45)	0.67		
Hypertension	1.01 (0.36 to 2.84)	0.97		
Diabetes mellitus	0.77 (0.14 to 4.18)	0.77		
Proteinuria (g/g)	1.35 (0.86 to 2.12)	0.13		
Urinary uromodulin/Cr/10 (mg/g)	0.93 (0.88 to 0.99)	0.0062	0.96 (0.90 to 1.03)	0.24
Hb (g/dl)	0.93 (0.70 to 1.23)	0.62		
Alb (g/dl)	1.97 (0.91 to 4.23)	0.08		
Cr (mg/dl)	3.42 (1.44 to 8.15)	< .0001	1.85 (0.62 to 5.56)	0.22
Serum uromodulin/10 (ng/ml)	0.83 (0.76 to 0.93)	< .0001	0.78 (0.66 to 0.93)	0.0008
CRP (mg/dl)	0.95 (0.87 to 1.04)	0.24		
MPO-ANCA titer (IU/ml)	1.00 (0.99 to 1.00)	0.69		

Abbreviations: HR, hazard ratio; CI, confidence interval; Cr, creatinine; Hb, hemoglobin; CRP, C-reactive protein; MPO, myeloperoxidase; ANCA, anti-neutrophil cytoplasmic antibody.

Each parameter that was significant in univariable analysis, was analyzed in a multivariable analysis.

## Discussion

It is important to detect biomarkers that can provide prognostic or pathological information independent of conventional factors such as Cr or eGFR in AAG patients, for whom it is often difficult to perform renal biopsy because of advanced age, weakness, or pulmonary hemorrhage. sUMOD is a promising circulating marker that might have prognostic value in kidney diseases, including CKD [14, 15], AKI [17], and diabetic kidney disease (DKD) [23]. Recently, Bjornstad et al. reported that higher baseline sUMOD levels predicted lower odds of incident DKD over 12 years in adults with type 1 diabetes [23]. Hence, it is of great interest to evaluate the clinical significance of sUMOD in AAG patients. Our data demonstrated that sUMOD levels in AAG patients were negatively correlated with serum Cr levels and positively correlated with eGFR. In contrast, there was no significant relationship between sUMOD levels and proteinuria. In addition, low sUMOD levels were associated with severe histopathological classes, which had poor renal prognosis compared to the focal class, and this association remained significant even after adjusting for serum Cr and uUMOD. To the best of our knowledge, this is the first study that has evaluated the clinical significance of sUMOD levels in AAG patients.

There are currently no reports regarding the relationship between renal histopathological findings and the levels of sUMOD. We first demonstrated that low levels of sUMOD at the time of renal biopsy reflected the severity of renal histopathological findings and poor renal prognosis in AAG patients. There was a significant relationship between sUMOD levels and the percentage of focal class, crescentic class, and mild tubulointerstitial lesions. Multivariate analyses revealed that low sUMOD predicted severe histopathological classes independent of serum Cr. The sUMOD levels in the severe histopathological classes were significantly lower compared to the focal class. In addition, there are currently no reports evaluating the correlation between sUMOD and uUMOD in patients with kidney diseases. We found a positive correlation between sUMOD and uUMOD, and both of them corelated with renal function in AAG patients. However, low uUMOD was not an independent risk factor for severe histopathological classes in AAG patients.

The decline of sUMOD in AAG patients with renal dysfunction can be attributed to the disturbed production of UMOD by renal tubular cells. In fact, most cases of AAG have tubulointerstitial injury, which can influence UMOD production. Our study demonstrated that sUMOD levels in AAG patients were associated with the severity of tubulointerstitial injury. We then examined the correlation between sUMOD and proximal tubular injury markers. There was no correlation between sUMOD and NAG index or urinary β2-MG, which may be explained by the fact that the source of circulating UMOD comes from epithelial cells lining the TAL of the loop of Henle, and not proximal tubules. Some other very promising urinary biomarkers for AKI, including kidney injury molecule-1 (KIM-1) and neutrophil gelatinase-associated lipocalin (NGAL), have shown no significant ability to distinguish active renal disease from remission in AAG [24]. UMOD may be a sensitive biomarker reflecting the Henle loop injury induced by crescentic glomerulonephritis or peritubular capillaritis in AAG. However, one of the main functions of UMOD is to protect against urinary tract infection (UTI) [25]. Uromodulin binds to type 1 fimbriae of *Escherichia coli* and thereby blocks colonization of urothelial cells [26]. UMOD interacts with other molecules and cells including cytokines [27], IgG [13, 28], complements [29, 30], neutrophils [31], T cells [32], and monocytes [30]. However, the immunomodulatory effects of UMOD in AAG are unclear, because there was no correlation between sUMOD and CRP or MPO-ANCA as immunological parameters in the present study.

We excluded patients who had not undergone renal biopsy examination. In 2010, Berden et al. reported a new classification for AAG, and the four categories (focal, crescentic, mixed, and sclerotic) significantly correspond to renal survival [18]. The focal class, containing ≥50% of normal glomeruli, has the best renal survival, while the sclerotic class, containing ≥50% of global sclerotic glomeruli, has the worst renal survival among the categories. These results have been reproduced in other clinical studies [19, 21]. In this study, the renal survival of the focal class was 100% during our observation period, which was the best survival rate. However, contrary to our expectation, the renal survival of the sclerotic class was better than the crescentic class (80% vs. 38%). This discrepancy might be due to population heterogeneity and the influence of immunosuppressive therapy on renal histopathological findings. In this study, all patients were MPO-ANCA positive, and most patients were diagnosed with MPA (90%). In addition, 46% of renal biopsies were performed after the initiation of immunosuppressive therapy.

The major limitations of this study are its retrospective design, relatively small sample size in a single-center analysis, and including patients after immunosuppressive treatment. A baseline comparison of the pre-treatment group and the post-treatment group showed that TP was significantly low (6.9 ± 0.9 vs. 6.4 ± 0.7; P = 0.011), and WBC was high (8533 ± 3708 vs. 11533 ± 3619, P = 0.002) in the post-treatment group. These reasons might be due to numbers of patients with poor nutritional status and the effect of steroid treatment. No significant differences were observed in other clinical findings, such as Cr (1.9 ± 1.5 vs. 2.1 ± 1.6; P = 0.61), proteinuria (1.4 ± 1.9 vs. 1.0 ± 1.1; P = 0.22), sUMOD (156.6 ± 70.0 vs. 6.4 ± 0.7; P = 0.35), uUMOD (163.6 ± 167.1 vs. 185.8 ± 122.5, P = 0.31) and distribution of histopathological classes between the groups. However, it is uncertain how much immunosuppressive treatment affected UMOD levels and renal function. These limitations should be addressed in future study.

In the present study, high sUMOD correlated with focal class independent of serum Cr. For now, sUMOD is a very useful clinical parameter for predicting the severity of histopathological findings. Several studies have shown that sUMOD levels are clearly correlated with renal function, and they decline along with the progression of CKD [14–16]. Of note, sUMOD levels are superior in their ability to distinguish between non-CKD and CKD stage 1 with eGFR >90 ml/min compared to serum Cr or eGFR, suggesting that sUMOD levels reflect CKD beyond the range detectable by Cr and the structural integrity of distal nephron masses [15]. Cr and eGFR are classical parameters that are influenced by muscle mass or diet and reflect renal function in terms of filtration ability, whereas sUMOD may reflect total kidney function. Interestingly, it has been reported that lower sUMOD is associated with greater risk for kidney allograft failure [33, 34].

In conclusion, low sUMOD levels were associated with severe clinicopathological findings and might be considered as a risk factor of progressive renal dysfunction in AAG. We should investigate the exact mechanisms involved in this phenomenon and explore the potential use of sUMOD levels as a novel prognostic biomarker in AAG for prospective analysis.

## Supporting information

S1 Dataset Original data. (XLSX)
